# Initial treatment and resource utilization among patients with metastatic-castration sensitive prostate cancer in Japan: a retrospective real-world study

**DOI:** 10.1093/jjco/hyae177

**Published:** 2024-12-20

**Authors:** Takahiro Kimura, Takuma Ito, Tomoyuki Taguchi, Kana Hattori, Rei Matsuyama

**Affiliations:** Department of Urology, The Jikei University School of Medicine, Tokyo, Japan; Market Access & Public Affairs, Bayer Yakuhin, Ltd., Osaka, Japan; Market Access & Public Affairs, Bayer Yakuhin, Ltd., Osaka, Japan; Medical Affairs & Pharmacovigilance, Bayer Yakuhin, Ltd., Osaka, Japan; Market Access & Public Affairs, Bayer Yakuhin, Ltd., Osaka, Japan; Medical Affairs & Pharmacovigilance, Bayer Yakuhin, Ltd., Osaka, Japan; Market Access & Public Affairs, Bayer Yakuhin, Ltd., Osaka, Japan

**Keywords:** treatment, resources, metastatic-castration sensitive prostate cancer, Japan

## Abstract

**Objectives:**

The introduction of novel drugs for metastatic castration-sensitive prostate cancer has expanded treatment options for patients. Associated changes in healthcare resource utilization may have occurred in tandem, but nationwide information is limited. This study aimed to describe initial treatment patterns and healthcare resource utilization (including costs) for patients with metastatic castration-sensitive prostate cancer in routine clinical practice in Japan.

**Methods:**

This retrospective, longitudinal cohort study used a large-scale claims database covering acute care hospitals of various sizes. Included were men who received first medical treatment for metastatic castration-sensitive prostate cancer between January 2015 and July 2021 (identification period). The primary endpoint was the initial treatment pattern for metastatic castration-sensitive prostate cancer.

**Results:**

Among 7665 men with metastatic castration-sensitive prostate cancer, the median (Q1, Q3) duration of first-line therapy was 8.2 (3.4, 17.3) months. During the overall period between 2015 and 2021, the most common initial pharmacotherapy (88.1% of treatment regimens) was ‘combined androgen blockade or androgen deprivation therapy only or first-generation anti-androgen only’. Use of androgen receptor signaling inhibitors increased following their introduction in 2018, reaching 26.6% of treatments started in 2021 (abiraterone + androgen deprivation therapy 9.4%, apalutamide + androgen deprivation therapy 9.2%, enzalutamide + androgen deprivation therapy 8.0%). Median total healthcare-related cost per person-year was JPY 244 479, with metastatic castration-sensitive prostate cancer drugs accounting for approximately one-third of the cost (JPY 396 620).

**Conclusions:**

Since androgen receptor signaling inhibitors were introduced, treatment patterns in patients with metastatic castration-sensitive prostate cancer in Japan have shifted, with an increased trend toward prescription of these agents. However, the most frequently used regimen for first-line treatment continues to be ‘combined androgen blockade or androgen deprivation therapy only or first-generation anti-androgen only’.

## Teaser

A real-world database analysis showed that, while prescription of androgen receptor signaling inhibitors has increased, combined androgen blockade or androgen deprivation therapy remains the most common regimen for initial treatment of metastatic castration-sensitive prostate cancer patients in Japan.

## Introduction

Prostate cancer is the most common type of cancer among Japanese men [1]. In 2023, there were an estimated 98 600 new cases of prostate cancer in Japan and it was the sixth most common cause of cancer deaths [1]. More than 10% of patients with prostate cancer have evidence of metastases at first diagnosis [2,3]. As prostate cancer is driven by androgens, most tumours respond initially to androgen deprivation through medical or surgical castration [2]. Effective treatment of metastatic castration-sensitive prostate cancer (mCSPC) is crucial for delaying disease progression [4].

The treatment paradigm for mCSPC has undergone a substantial change over the last decade. While androgen deprivation therapy (ADT) continues to be the backbone of treatment, survival is improved by adding docetaxel and/or an androgen-receptor signaling inhibitor (ARSI) [2–7]. ADT plus ARSI doublet therapy is now considered to be standard of care for patients with mCSPC worldwide, although further intensification may be appropriate in some patients [2–7]. In Japan, ARSIs (abiraterone, enzalutamide, and apalutamide) have become successively available since 2018 as options to combine with ADT in the treatment of mCSPC [3]. Docetaxel was added as an option in 2021, and triplet therapy comprising docetaxel and ADT plus darolutamide (ARSI) was launched in 2023.

Owing to the high cost of ARSIs, concerns have been raised about the shifting treatment patterns for patients with mCSPC and associated financial burden [8,9]. Moreover, the lack of clear agreement about the optimal choice of agents for initial therapy creates challenges for clinicians as regards treatment selection [4,10,11]. Treatment patterns, healthcare resource burden, and costs for managing patients with mCSPC remain poorly studied in Japan. Gaining an understanding of the current treatment of patients with mCSPC in real-world clinical practice is recognized as an important step towards defining the best fit for newer therapies within the treatment paradigm. This study aimed to clarify these uncertainties by describing initial treatment patterns and associated healthcare resource utilization (HCRU) for patients with mCSPC managed in routine clinical practice in Japan.

## Patients and methods

### Study design

This retrospective, longitudinal cohort study used a large-scale real-world claims database provided by Medical Data Vision (MDV) Co, Ltd. (Tokyo, Japan). The data source period for the study was 01 April 2008 to 31 July 2022.

The MDV database is based on routinely collected claims data derived from acute care hospitals participating in the Diagnosis Procedure Combination (DPC) system in Japan [12]. Across Japan, MDV covers ~25% of DPC hospitals of various sizes based on bed numbers. DPC claims data include inpatient and outpatient records, prescribed drugs, diagnoses, laboratory tests performed, and laboratory test results for a proportion of samples.

### Study population

The study population comprised men with mCSPC (diagnosed at the same time as the initial prostate cancer diagnosis or those with disease recurrence).

Study participants were identified by searching the MDV database for men with a confirmed diagnosis of metastatic prostate cancer during the data source period of 01 April 2008 to 31 July 2022 and excluding those who had been diagnosed with castration-resistant prostate cancer (CRPC; disease code 8848040) at the time of diagnosis. Metastatic prostate cancer was defined as: (i) patients with a confirmed diagnosis of prostate cancer (ICD-10: C61) and a confirmed diagnosis of secondary malignant neoplasm (ICD-10: C77–C79) after the prostate cancer diagnosis. The date of the first diagnosis of secondary malignant neoplasm was taken as the diagnosis date for metastatic prostate cancer; and/or (ii) patients with a confirmed diagnosis of prostate cancer with ‘M1’ status (UICC TNM Classification). The date of diagnosis of prostate cancer was taken as the diagnosis date for metastatic prostate cancer.

An identification period within the full data source period was defined as 01 January 2015 to 31 July 2021 to account for the establishment of a disease code for CRPC in July 2014 after enzalutamide and abiraterone were covered by health insurance, and to allow for follow-up before the end of the full period (31 July 2022). Inclusion criteria required that patients have a record of ‘index treatment’ (the first prescription after a diagnosis of metastatic prostate cancer) during the identification period. A further criterion was for patients to have a confirmed diagnosis of prostate cancer (ICD-10: C61 ‘malignant neoplasm of the prostate’) during a look-back period of 6 months prior to the index treatment date. Patients who received ADT by medication during the follow-up period were included.

Patients were excluded if they had a code for CRPC before the index date or if they had another primary malignancy (ICD-10: C00-C97, except C61 and C77-C79) that was not in remission, within 5 years before the mCSPC diagnosis.

The index date was defined as the date of a first prescription record for ADT, docetaxel or ARSI after the mCSPC diagnosis, without any eligible drugs for mCSPC and/or drugs for metastatic castration-resistant prostate cancer (mCRPC) recorded during the prior 6-month period.

Patients’ records were followed from the index date to the earliest of specified timepoints: end of data source period, diagnosis of mCRPC, date of in-hospital death, or last recorded patient record.

### Endpoints

The primary endpoint was the initial treatment pattern for mCSPC patients in Japan. This included patient characteristics, proportion of patients per treatment type, and treatment duration. Specifically, the study aimed to describe the proportion of mCSPC patients who received the following treatments as initial treatment after a diagnosis of mCSPC: abiraterone + ADT; docetaxel + ADT; enzalutamide + ADT; apalutamide + ADT; other chemotherapies; combined androgen blockade (CAB) or ADT only or first-generation anti-androgen only; or any other treatment combinations. All eligible drugs prescribed within 90 days after the index date were considered initial treatment for mCSPC.

Secondary endpoints were HCRU outcomes (utilization rates and costs) and treatment outcomes. HCRU rates per person-year included number and length of inpatient stays, number of outpatient visits, number of emergency room (ER) visits, and number of radiation therapy sessions. HCRU costs per person-year included total cost, cost of inpatient admissions, cost of outpatient visits, cost of drugs (all drugs, mCSPC cancer drugs, other drugs), and costs for scans (positron emission tomography, computed tomography, magnetic resonance imaging), rectal examinations, biopsies and radiation therapy. A further analysis examined HCRU costs per person-year according to calendar year (of index date) during the identification period. Treatment outcomes were the time-to-event endpoints of time from index date to mCRPC diagnosis and time from index date to prostate-specific antigen (PSA) progression, with the latter defined according to Prostate Cancer Clinical Trials Working Group 3 criteria.

### Statistical analysis

Data were analysed descriptively using summary statistics for quantitative (continuous) and categorical data. Continuous data were described by the number of non-missing values, mean and standard deviation (SD) if the data showed a symmetrical distribution or by median and lower and upper quartiles (Q1, Q3) if otherwise. Frequency tables were generated for categorical data.

Time-to-event outcomes were analysed using Kaplan–Meier curves and their medians (95% confidence intervals). For each time-to-event outcome, patients without an event of interest during the period of interest were considered censored.

All data analyses were performed using the SAS software package version 9.4 or higher (SAS Institute Inc., Cary, NC, USA).

## Results

**Figure 1:**
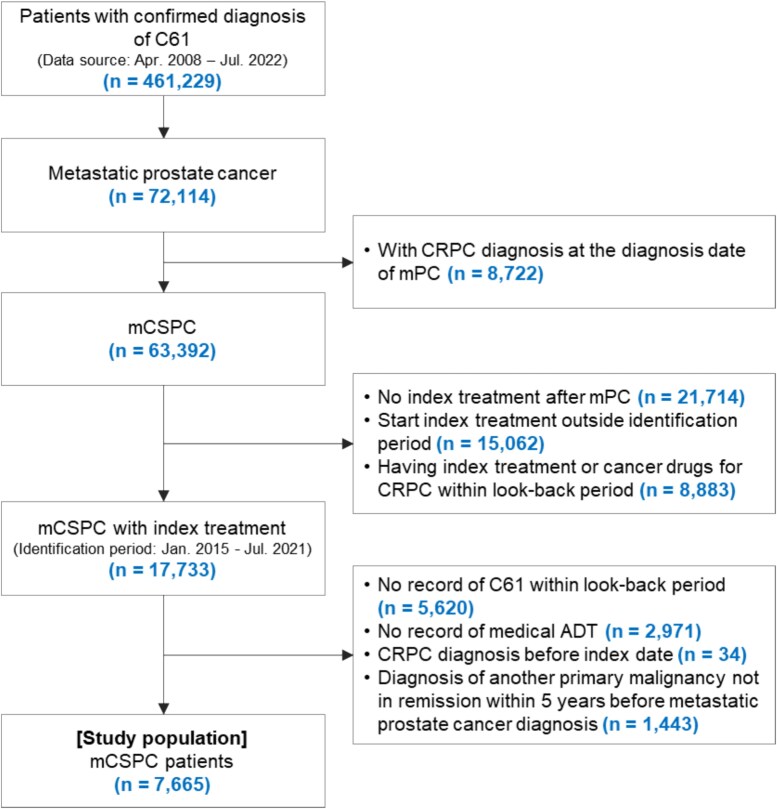
Patient disposition. ADT, androgen deprivation therapy; C61, ICD-10 code C61 ‘malignant neoplasm of the prostate’; CRPC, castration-resistant prostate cancer; mCSPC, metastatic castration-sensitive prostate cancer; mPC, metastatic prostate cancer.

Among 72 114 men with a confirmed diagnosis of metastatic prostate cancer between 01 April 2008 and 31 July 2022, 63 392 had mCSPC. Of these, 7665 men met all inclusion criteria and were included in the analysis population (Fig. 1).

### Baseline characteristics

Baseline characteristics are summarized in Table 1. The mean (SD) age of the mCSPC study population at baseline was 75.3 (8.4) years. Median (Q1, Q3) duration of follow-up was 17.1 (7.3, 33.7) months. Common comorbidities were congestive heart failure (9.2%), followed by mild liver disease (6.9%) and chronic pulmonary disease (6.5%).

**Table 1:** Baseline demographic and clinical characteristics of the study population.

Variable	All mCSPC patients (n = 7665)
Age, years, mean (SD)	75.3 (8.4)
Age group, years, n (%) < 65 ≥ 65	726 (9.5) 6,939 (90.5)
Weight, kg, mean (SD)	60.7 (10.9)
Index year, n (%) 2015 2016 2017 2018 2019 2020 2021	819 (10.7) 968 (12.6) 1090 (14.2) 1381 (18.0) 1403 (18.3) 1376 (18.0) 628 (8.2)
Modified CCI comorbidities, n (%) Congestive heart failure Mild liver disease Chronic pulmonary disease Renal disease Diabetes with chronic complications Dementia Hemiplegia or paraplegia Rheumatological disease Moderate or severe liver disease HIV	706 (9.2) 528 (6.9) 499 (6.5) 399 (5.2) 230 (3.0) 176 (2.3) 133 (1.7) 85 (1.1) 19 (0.2) 3 (0.03)
Modified CCI scores, median (Q1, Q3)	6.0 (6.0, 7.0)
Modified CCI score category, n (%) 6 7 8 9+	5654 (73.8) 578 (7.5) 904 (11.8) 529 (6.9)
Duration of follow-up, months, median (Q1, Q3)	17.1 (7.3, 33.7)

CCI, Charlson Comorbidity Index; mCSPC, metastatic castration-sensitive prostate cancer; Q, quartile; SD, standard deviation.

### Treatment pattern of patients with mCSPC

Among the study population, 59.8% of patients received a single line of therapy (Table 2). Median (Q1, Q3) duration of first-line therapy was 8.2 (3.4, 17.3) months, and 21.4% of patients received first-line therapy lasting <90 days.

**Table 2:** Initial treatment for mCSPC.

Variable	All mCSPC patients (n = 7665)
Number of lines of therapy, n (%) 1 2+	4585 (59.8) 3080 (40.2)
Treatment duration for first line of therapy Median (Q1, Q3)	8.2 (3.4, 17.3)
Patients with a treatment line lasting <90 days, n (%)	1641 (21.4)
Treatment regimen in the first line of therapy, n (%) ADT only OR 1st generation anti-androgen only OR ADT with 1st generation anti-androgen Abiraterone + ADT Docetaxel + ADT Enzalutamide + ADT Apalutamide + ADT Other chemotherapy Any other treatment combinations	6755 (88.1) 382 (5.0) 140 (1.8) 124 (1.6) 125 (1.6) 78 (1.0) 61 (0.8)
Patients who underwent radiation therapy during follow-up, n (%) All During first line of therapy	1129 (14.7) 800 (11.5)
Patients who underwent surgical castration during follow-up, n (%) All During first line of therapy	289 (3.8) 167 (2.2)

ADT, androgen deprivation therapy; mCSPC, metastatic castration-sensitive prostate cancer; Q, quartile.

The most common initial treatment (88.1% of treatment regimens) for patients with mCSPC was ‘CAB or ADT only or first-generation anti-androgen only’ (Table 2). This was followed by abiraterone + ADT (5.0%). During first-line treatment, 11.5% of patients also received radiation therapy, and 2.2% underwent surgical castration.

**Figure 2:**
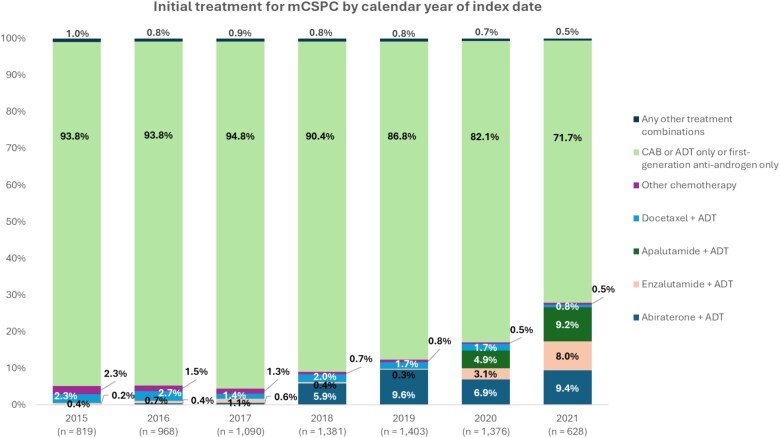
Annual trends in initial treatment for mCSPC. Data indicate the percentage of patients receiving each regimen as initial treatment according to calendar year (of index date). The identification period for 2021 was 7 months (January to July). ADT, androgen deprivation therapy; CAB, combined androgen blockade; mCSPC, metastatic castration-sensitive prostate cancer.

Prescribing of ARSIs increased over time, reaching ~27% in patients who started initial treatment for mCSPC in 2021 (Fig. 2). Among available ARSIs, abiraterone + ADT was the most frequently used regimen (9.4%), followed by apalutamide + ADT (9.2%) and enzalutamide + ADT (8.0%). Chemotherapy with docetaxel was used in ≤2% of patients who started initial treatment for mCSPC during the last 5 years of the identification period.

### Healthcare resource use among patients with mCSPC

During follow-up of patients with mCSPC, the median (Q1, Q3) number of inpatient admissions per person-year was 1.3 (0.6, 3.3) and the median (Q1, Q3) length of inpatient stay was 15.3 (4.0, 60.6) days (Table 3). The median number of outpatient visits was 13.6 (8.7, 19.8) per person-year. The median (Q1, Q3) number of ER visits was 0.9 (0.4, 1.8) per person-year, and the median (Q1, Q3) number of radiation therapy sessions as part of initial treatment was 17.2 (7.5, 37.3) per person-year.

**Table 3:** Healthcare resource utilization rates per person-year during follow-up of patients with mCSPC.

Variable	All mCSPC patients (n = 7665)
Number of inpatient admissions [n = 4655] Median (Q1, Q3)	1.3 (0.6, 3.3)
Length of inpatient stay (days) [n = 4654] Median (Q1, Q3)	15.3 (4.0, 60.6)
Number of ICU admissions [n = 215] Median (Q1, Q3)	0.46 (0.26, 0.95)
Number of outpatient visits [n = 7163] Median (Q1, Q3)	13.6 (8.7, 19.8)
Number of ER visits [n = 295] Median (Q1, Q3)	0.9 (0.4, 1.8)
Number of radiation therapy sessions [n = 1129] Median (Q1, Q3)	17.2 (7.5, 37.3)

ER, emergency room; ICU, intensive care unit; mCSPC, metastatic castration-sensitive prostate cancer; Q, quartile.

During follow-up of patients with mCSPC, the median (Q1, Q3) total healthcare-related cost per person-year was JPY 244 479 (627 993,170 454). The median cost of mCSPC cancer drugs accounted for approximately one-third of the total cost (JPY 396 620) (Table 4).

**Table 4:** Healthcare resource utilization costs per person-year during follow-up of patients with mCSPC.

Variable	All mCSPC patients (n = 7665)
Total cost [n = 7665] Median (Q1; Q3)	1 244 479 (627 993; 3,170 454)
Cost of inpatient admissions [n = 4655] Median (Q1; Q3)	810 893 (245 446; 2 731 913)
Cost of outpatient visits [n = 7163] Median (Q1; Q3)	697 807 (447 728; 1 212 449)
Cost of all drugs [n = 7665] Median (Q1; Q3)	603 744 (357 741;191 774)
Cost of mCSPC cancer drugs [n = 7665] Median (Q1; Q3)	396 620 (274 926; 611 146)
Cost of other drugs^a^ [n = 7305] Median (Q1; Q3)	138 682 (37 421; 470 658)
Cost for PET scan [n = 359] Median (Q1; Q3)	26 585 (11 087; 63 386)
Cost for CT scan [n = 5778] Median (Q1; Q3)	20 718 (10 678; 40 264)
Cost for MRI scan [n = 2548] Median (Q1; Q3)	11 931 (6103; 25 170)
Cost for rectal examinations [n = 36] Median (Q1; Q3)	3366 (1461; 6229)
Cost for biopsies [n = 710] Median (Q1; Q3)	13 510 (6791; 32 674)
Cost for radiation therapy [n = 1129] Median (Q1; Q3)	230 684 (96 938; 494 855)

CT, computed tomography; mCSPC, metastatic castration-sensitive prostate cancer; MRI, magnetic resonance imaging; PET, positron emission tomography; Q, quartile.

^a^All drugs except mCSPC cancer drugs.

In the analysis of HCRU costs per person-year according to calendar year (of index date), between 2015 and 2021 the median cost rose for inpatient admission (from JPY 645 194 to JPY 319 748) and radiation therapy (from JPY 99 556 to JPY 576 675). Conversely, the median cost of anti-cancer drugs for mCSPC, even after the introduction of ARSI, was lower in 2021 (JPY 400 633) than in 2015 (JPY 464 712) (Supplementary Table S1).

### Time-to-event outcomes

Because PSA test values were available for only around 10% of patients, an overall evaluation was not possible. However, to verify the possibility of extrapolating the results to the patients included in this study, a Kaplan–Meier analysis was performed on time to PSA progression and time to CRPC.

The median (Q1, Q3) time to PSA progression was 21.7 (19.0, 26.1) months. The median (Q1, Q3) time from the index date for mCSPC treatment to a diagnosis of mCRPC was 54.3 (51.1, 62.9) months (Supplementary Fig. 1).

## Discussion

To the best of our knowledge, this is the first study to analyse first-line treatment patterns and HCRU for patients with mCSPC based on a large-scale, real-world claims database derived from various-sized DPC hospitals across Japan [12]. As the treatment landscape for mCSPC has changed rapidly in recent years, our analysis of changes in treatment patterns over time provides a valuable baseline for future research.

In this study, we analysed administrative data for 7665 patients who began medical treatment for mCSPC between January 2015 and July 2021. The mean age of the cohort (75 years) was representative of the mCSPC population as per studies based on Japanese registry data [13,14]. Among patients with available data, the median time to PSA progression (21.7 months) aligned broadly with that reported in phase 3 clinical trials of mCSPC: 7.4–44 months with ADT alone [15,16] and 33.2 months with abiraterone + ADT [15]. The median time to mCRPC diagnosis (54.3 months) exceeded the median time to PSA progression, likely because of cases where CRPC had not been diagnosed at the time of PSA progression. CRPC diagnostic criteria specify that the PSA value must increase three times in a row with a measurement interval of at least one week, and with two increases of 50% or more from the lowest value [17].

Although combination therapy with ARSI + ADT is widely used in many countries, our analysis revealed that 88% of patients with mCSPC in Japan between January 2015 and July 2021 received ‘CAB or ADT only or first-generation anti-androgen only’ as first-line therapy. This finding is consistent with a report of the IPSOS Global Oncology Monitor database during the period January 2018 to June 2020 which described a 76% usage rate of non-guideline-concordant therapies [18]. Regarding ARSI + ADT combination therapy in Japan, abiraterone has been reimbursed for high-risk mCSPC since 2018, and enzalutamide and apalutamide have been reimbursed for mCSPC since 2020. Reflecting this change in reimbursement status, the annual trend analysis revealed an upward shift in the proportion of patients who received ARSI + ADT as their first-line treatment from 6% in 2018 to 27% in 2021, with a corresponding decrease in the use of ‘CAB or ADT only or first-generation anti-androgen only’ therapy. A recent survey of 38 Japan Clinical Oncology Group-affiliated hospitals covering the period April 2022 to March 2023 reported that 56% of patients received ARSI + ADT as the first-line treatment for mCSPC [9]. The lower usage rate we reported may have been influenced by differences in target institutions between the studies. Overall, the trend points to an increase in ARSI prescriptions. This is relevant clinically in light of a retrospective analysis in Japanese patients with mCSPC which identified a significantly longer castration-resistant prostate cancer-free survival time between patients treated with upfront ARSI and those treated with CAB (median 36.7 vs. 12.3 months, hazard ratio: 0.44, 95% confidence interval: 0.20–0.97, *P* = 0.035) [19].

Docetaxel + ADT (upfront docetaxel) is also a common intensive first-line therapy for mCSPC. We found that relatively few patients with mCSPC in our cohort received upfront docetaxel, which may reflect differences in the reimbursement status of available options. Based on outcomes reported in the STAMPEDE and CHAARTED trials [20–22], the efficacy of upfront docetaxel has been widely recognized in many countries. In Japan, the indication for docetaxel in prostate cancer was initially limited to CRPC, and then expanded to mCSPC in 2021, which was after ARSI were approved to manage mCSPC. As such, the identification period of January 2015 and July 2021 in the current analysis was not sufficiently long to observe an increase in the number of patients treated with docetaxel. In 2023, triplet therapy with darolutamide, docetaxel and ADT for mCSPC was approved in Japan based on the ARASENS trial [23]. Trends in intensive therapy with upfront docetaxel-containing regimens in mCSPC continue to be of interest.

As the number of prostate cancer cases in Japan is expected to increase with population ageing [24], there is growing concern about the impact on future healthcare costs of a continued shift towards intensified regimens. A survey of Japan Clinical Oncology Group-affiliated hospitals reported that monthly drug costs for the treatment of mCSPC were 12.8–25.9 times higher for ADT + ARSI (depending on the ARSI) than for ADT alone or CAB [9]. Our analysis based on claims data from 2015 to 2022 indicated that the major contributor to total annual healthcare costs (median JPY 244 479 per person-year) for our cohort with mCSPC was the costs associated with hospital admissions (median 1.3 hospitalizations per person year), while the cost of anti-cancer drugs for mCSPC represented approximately one-third of the total. Notably, the annual trend analysis we undertook demonstrated that the cost of anti-cancer drugs for mCSPC after the introduction of ARSI has not yet reached a level that would elevate median overall healthcare and drug costs. It would be beneficial to continue monitoring changes in healthcare cost breakdowns, as well as conduct a cost-effectiveness analysis that encompasses not only the cost of drugs, but also the overall costs associated with treatment, including testing, hospitalizations and management of side effects and complications.

As a secondary analysis of administrative health data derived from acute care hospitals participating in the DPC system, this study has limitations. Patients treated at institutions not included in the MDV database were not captured as part of the study population. The study duration is shorter than that of other observational studies because first-line treatment was defined as having changed when a new drug was added or the type of initial therapy was changed >90 days after the index date, even if the new drug had the same efficacy. Any prescriptions added >90 days after the index date are reflected in second- or later-line treatment. As patients who moved to other medical institutions could not be tracked, costs and resource utilization may have been underestimated. Lastly, because laboratory test results, including PSA levels, were available for a relatively small proportion (~10%) of patients in the database as is usual with claims data, progression to a mCRPC diagnosis may have been underestimated.

In conclusion, this analysis of a nationwide large-scale claims database between 2015 and 2022 in Japan shows that ‘CAB or ADT only or first-generation anti-androgens only’ continue to be the most frequently used treatment regimens for first-line therapy of patients with mCSPC. Since 2018, ARSIs have been increasingly prescribed. Future studies are warranted to investigate treatment patterns and economic implications as the use of ARSIs becomes more prevalent across Japan.

## Supplementary Material

JJCO_Submission_mCSPC_Japan_Supplement_Figure_011124_hyae177

JJCO_Submission_mCSPC_Japan_Supplement_Table_011124_hyae177

## Data Availability

The MDV database is commercially available from Medical Data Vision Co., Ltd.
