# Supplementary material for: Initial treatment and resource utilization among patients with metastatic-castration sensitive prostate cancer in Japan: a retrospective real-world study
Source: Jpn J Clin Oncol. 2024 Dec 20;55(4):399–405. doi: 10.1093/jjco/hyae177 (PMC11973639; doi:10.1093/jjco/hyae177)

**Supplementary figures**

**Supplementary Figure 1.** Kaplan-Meier curves for time to PSA progression (top) and time to mCRPC diagnosis (bottom). This sensitivity analysis was undertaken to verify the possibility of extrapolating the results to the target population of the study. mCRPC, metastatic castration-resistant prostate cancer; PSA, prostate-specific antigen.


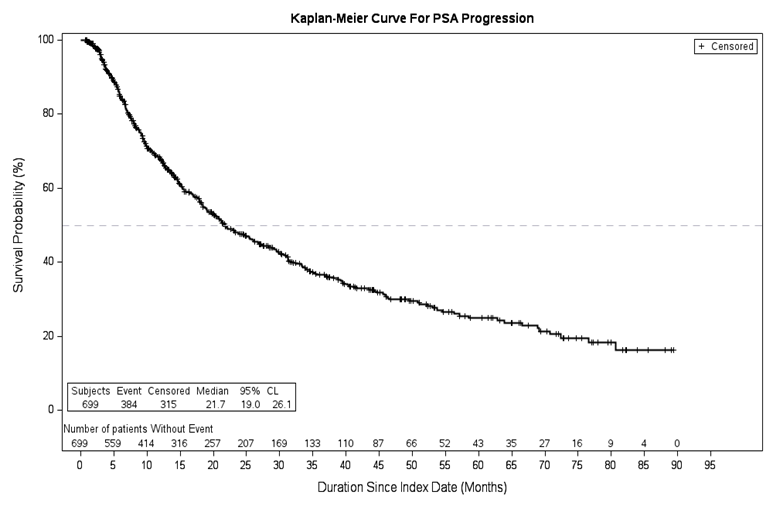


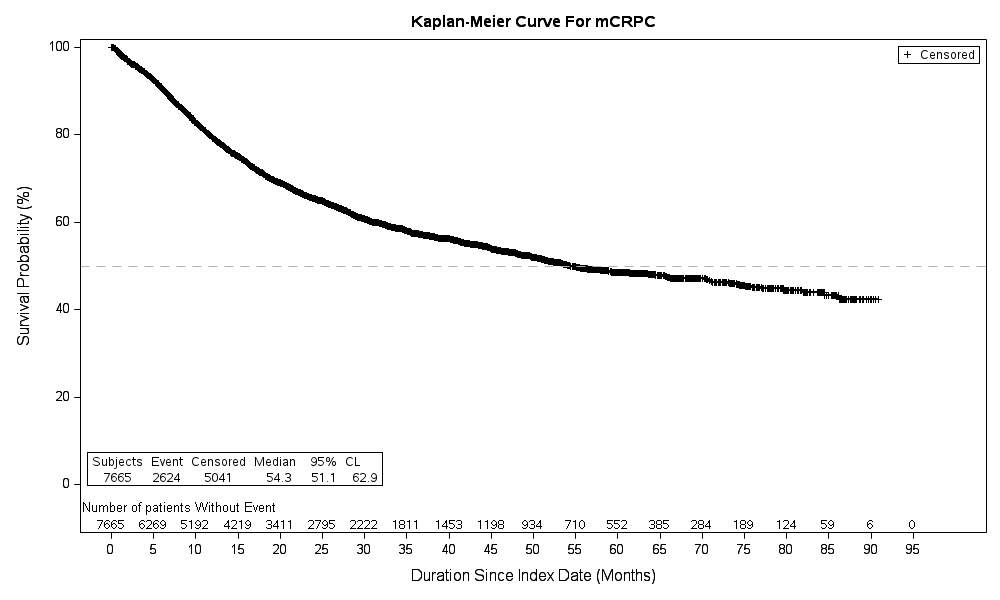

Supplement: JJCO_Submission_mCSPC_Japan_Supplement_Figure_011124_hyae177 [file jjco_submission_mcspc_japan_supplement_figure_011124_hyae177.docx]
