# Supplementary material for: Initial treatment and resource utilization among patients with metastatic-castration sensitive prostate cancer in Japan: a retrospective real-world study
Source: Jpn J Clin Oncol. 2024 Dec 20;55(4):399–405. doi: 10.1093/jjco/hyae177 (PMC11973639; doi:10.1093/jjco/hyae177)
Supplement: JJCO_Submission_mCSPC_Japan_Supplement_Table_011124_hyae177 [file jjco_submission_mcspc_japan_supplement_table_011124_hyae177.docx]

# Supplementary tables

**Supplementary Table S1**. Healthcare resource utilization costs per person-year according to calendar year (of index date) during follow-up of patients with mCSPC.

| Variable | Overall  (N = 7,665) | 2015  (N = 819) | 2016  (N = 968) | 2017  (N = 1,090) | 2018  (N = 1,381) | 2019  (N = 1,403) | 2020  (N = 1,376) | 2021  (N = 628) |
| --- | --- | --- | --- | --- | --- | --- | --- | --- |
| *n*  Total cost  Median (Q1; Q3) | *n* = 7,665  1,244,479  (627,993; 3,170,453) | *n* = 819  1,297,410  (705,168; 2,619,404) | *n* = 968  1,216,368  (658,112; 2,530,439) | *n* = 1,090  1,143,000  (603,470; 2,645,644) | *n* = 1,381  1,161,478  (609,077; 2,851,179) | *n* = 1,403  1,217,994  (599,977; 3,271,257) | *n* = 1,376  1,319,748  (616,111; 868,993) | *n* = 628  1,917,888  (685,216; 4,760,073) |
| *n*  Cost of inpatient admission  Median (Q1; Q3) | *n* = 4,655  810,893  (245,446; 2,731,913) | *n* = 558  645,194  (214,794; 2,457,526) | *n* = 639  727,017  (228,718; 2,367,303) | *n* = 714  744,605  (216,127; 2,497,794) | *n* = 857  738,608  (224,965; 2,321,814) | *n* = 831  816,212  (247,378; 2,771,018) | *n* = 744  992,934  (299,215; 3,104,725) | *n* = 312  1,319,748  (420,782; 4,463,860) |
| *n*  Cost of outpatient visits  Median (Q1; Q3) | *n* = 7,163  697,807  (447,728; 1,212,449) | *n* = 760  773,944  (490,289; 1,189,183) | *n* = 907  711,664  (436,784; 1,157,926) | *n* = 1,023  657,139  (436,933; 1,044,682) | *n* = 1,288  669,532  (444,894; 1,138,205) | *n* = 1,306  675,372  (423,115; 1,184,822) | *n* = 1,289  702,289  (454,440; 1,418,642) | *n* = 590  867,187  (480,259; 2,723,971) |
| *n*  Cost of all drugs  Median (Q1; Q3) | *n* = 7,665  603,744  (357,741; 1,191,774) | *n* = 819  701,639  (443,796; 1,208,581) | *n* = 968  642,023  (370,181; 1,112,239) | *n* = 1,090  574,248  (357,632; 1,016,953) | *n* = 1,381  564,563  (358,445; 1,128,104) | *n* = 1,403  561,608  (336,918; 1,139,445) | *n* = 1,376  567,595  (346,170; 1,424,384) | *n* = 628  744,010  (368,239; 2,590,854) |
| *n*  Cost of mCSPC cancer drugs  Median (Q1; Q3) | *n* = 7,665  396,620  (274,926; 611,146) | *n* = 819  464,712  (307,309; 633,264) | *n* = 968  421,621  (271,343; 595,848) | *n* = 1,090  398,601  (266,264; 553,103) | *n* = 1,381  390,017  (261,073; 554,556) | *n* = 1,403  373,727  (269,719; 592,171) | *n* = 1,376  364,894  (265,008; 776,166) | *n* = 628  400,633  (294,480; 2,051,100) |
| *n*  Cost of other drugs^a^  Median (Q1; Q3) | *n* = 7,305  138,682  (37,421; 470,658) | *n* = 799  192,896  (53,604; 538,588) | *n* = 942  148,246  (44,966; 487,797) | *n* = 1,061  135,005  (39,412; 452,335) | *n* = 1,320  150,671  (37,552; 484,795) | *n* = 1,321  128,898  (32,100; 438,329) | *n* = 1,285  110,136  (33,821; 434,050) | *n* = 577  110,354  (28,112; 475,083) |
| *n*  Cost for PET scans  Median (Q1; Q3) | *n* = 359  26,585  (11,087; 63,386) | *n* = 46  14,535  (8,531; 33,373) | *n* = 58  21,603  (7,675; 41,126 | *n* = 72  17,047  (9,667; 53,968) | *n* = 64  23,995  (7,701; 50,860) | *n* = 52  39,580  (26,299; 101,348) | *n* = 50  48,967  (16,193; 66,886) | *n* = 17  67,458  (25,816; 115,395) |
| *n*  Cost for CT scans  Median (Q1; Q3) | *n* = 5,778  20,718  (10,678; 40,264) | *n* = 652  17,586  (8,696; 38,034) | *n* = 783  19,490  (9,569; 37,569) | *n* = 872  19,035  (9,714; 40,088) | *n* = 1,014  20,824  (10,146; 40,088) | *n* = 1,043  20,137  (10,650; 41,929) | *n* = 992  23,348  (12,714; 42,919) | *n* = 422  25,344  (14,334; 44,251) |
| *n*  Cost for MRI scans  Median (Q1; Q3) | *n* = 2,548  11,931  (6,103; 25,170) | *n* = 291  9,013  (4,239; 18,331) | *n* = 346  9,697  (4,408; 19,868) | *n* = 399  10,469  (5,190; 23,023) | *n* = 503  11,015  (5,982; 22,700) | *n* = 432  12,166  (5,680; 27,328) | *n* = 394  15,323  (8,347; 30,552) | *n* = 183  19,707  (12,585; 34,615) |
| *n*  Cost for rectal examinations  Median (Q1; Q3) | *n* = 36  3,366  (1,461; 6,229) | *n* = 7  3,411  (1,695; 6,307) | *n* = 5  4,435  (998;  6,219) | *n* = 7  2,846  (1,189; 6,239) | *n* = 10  3,049  (1,388; 5,293) | *n* = 3  3,679  (3,320; 4,852) | *n* = 2  7,230  (1,665; 12,795) | *n* = 2  18,473  (5,060; 31,887) |
| *n*  Cost for biopsies  Median (Q1; Q3) | *n* = 710  13,510  (6,791; 32,674) | *n* = 80  7,570  (3,495; 15,590) | *n* = 85  10,026  (5,155; 21,306) | *n* = 96  11,277  (6,444; 24,200) | *n* = 130  18,105  (8,002; 54,399) | *n* = 131  16,131  (6,352; 33,205) | *n* = 124  13,247  (8,009; 57,462) | *n* = 64  14,804  (11,769; 54,200) |
| *n*  Cost for radiation therapy  Median (Q1; Q3) | *n* = 1,129  230,684  (96,938; 494,855) | *n* = 110  99,446  (46,302; 240,635) | *n* = 129  187,079  (103,911; 357,507) | *n* = 160  161,796  (104,390; 341,028) | *n* = 206  221,845  (94,529; 394,691) | *n* = 201  259,280  (94,694; 459,150) | *n* = 217  344,178  (144,148; 601,275) | *n* = 106  576,675  (178,378; 1,003,870) |
| ^a^ All drugs except mCSPC cancer drugs.  CT, computed tomography; mCSPC, metastatic castration-sensitive prostate cancer; MRI, magnetic resonance imaging; PET, positron emission tomography; Q, quartile. | | | | | | | | |
